# Loneliness, Complaining and Professional Burnout of Medical Personnel of Psychiatric Wards during COVID-19 Pandemic—Cross-Sectional Study

**DOI:** 10.3390/healthcare10010145

**Published:** 2022-01-13

**Authors:** Edyta Karcz, Agata Zdun-Ryżewska, Agnieszka Zimmermann

**Affiliations:** 1 Division of Neurological and Psychiatric Nursing, Faculty of Health Sciences Medical University of Gdańsk, 80-210 Gdańsk, Poland; 2 Department of Quality of Life Research, Faculty of Health Sciences, Medical University of Gdańsk, 80-210 Gdańsk, Poland; agata.zdun-ryzewska@gumed.edu.pl; 3 Department of Medical and Pharmaceutical Law, Faculty of Health Sciences, Medical University of Gdańsk, 80-210 Gdańsk, Poland; agnieszka.zimmermann@gumed.edu.pl

**Keywords:** complaining, medical staff, psychiatric, loneliness, occupational burnout

## Abstract

Background: Professional burnout in the medical community has been present for a long time, also among mental health professionals. The aim of the study was to examine the links between loneliness, complaining and professional burnout among medical personnel in psychiatric care during a pandemic. Loneliness and complaining of the medical staff are not documented in the literature well enough. Methods: Oldenburg Burnout Questionnaire, the Loneliness Scale, the Complaint Questionnaire and author’s questionnaire. The respondents: 265 medical employees—doctors (19.2%), nurses (69.8%), paramedics (4.9%), medical caregivers (5.7%). Results: Loneliness and complaining are significant predictors of exhaustion. The model explains 18% of exhaustion variance. Loneliness, complaining and job seniority are also predictors of disengagement; the model allows to predict 10% of the variance of disengagement. Women are more prone to complain. Complaining significantly correlates with direct support from management. A high rate of loneliness correlates, in a statistically significant way, with worse work organization, less management support, worse atmosphere in the team and with more irresponsible attitudes of colleagues. Conclusions: Loneliness and complaining can be used to predict occupational burnout. Women and people without management support complain more often. Loneliness is connected with bad work organization and bad cooperation in a team.

## 1. Introduction

The speed and extent of the spread of the pandemic COVID-19 has caused health and life threats to medical professionals on an unprecedented scale [1]. Numerous studies conducted since the onset of the presence of the virus indicate many factors that are a source of stress for staff. These are, among others: fear for their own safety, the risk of infecting members of their family and loved ones, but also team members [2,3], anxiety about the availability of personal protective equipment, and lack of adequate support [3,4].

An element that significantly enhances the intensity of the impact of the above factors on the mental health of employees is the chronicity of the situation. Functioning in such difficult conditions for a long period of time poses a very high risk to both physical and mental health of all people, in particular health care employees, who are probably more than before exposed to the phenomenon of occupational burnout.

Professional burnout is a response to the long-term effects of interpersonal stressors at work [5] and a form of occupational tension resulting from the buildup of work-related stress [6]. Work burnout commonly understood as a syndrome consisting of exhaustion defined as a consequence of intense physical and mental stress and disengagement from work as a result of long-term stress at work [7]. Work burnout is one of the major problems among mental health care employees [8] also among mental health professionals. A recently published meta-analysis shows the presence of exhaustion in 40% of medical staff in this sector, while 19% could be described with low levels of personal accomplishment related to disengagement from work [9]. Due to the worldwide pandemic, those numbers will probably grow.

An element that is significantly related to burnout is loneliness. Loneliness occurs when people experience a sense of loneliness and when, regardless of the number of contacts, they begin to lack meaningful social relationships. This subjective feeling of loneliness depends more on the quality of the relationship. It is a kind of dissonance between real and desired social relations [10]. The current epidemic situation and the related need to maintain social distance has worsened interpersonal relations, thus limiting social support, which may also contribute to the growing feeling of loneliness.

The problem of the presence of loneliness in the professional life of medical employees is not described in detail in the literature. In the last few years, there have been studies on the existence of loneliness mainly among doctors, where the authors state that loneliness is common in the work of doctors and is associated with burnout [10]. In a study involving 401 family doctors, the incidence of loneliness was 44.9%. The analysis of the results showed that physicians who experienced a greater sense of loneliness more often reported at least one of the symptoms of occupational burnout [11]. In other studies, loneliness has been identified as one of the leading stressors in the work of nursing managers [12].

The most common intention of complaining people is experiencing a sense of relief by expressing dissatisfaction, which, as most people seem, should have a soothing effect on unpleasant emotional states [13]. However, there are reports linking the phenomenon of burnout and complaints. Burnout is not just complaint, it is a much more serious condition, but it might be related to complaining [14].

Nowadays, protecting the health of medical employees providing services in the field of psychiatric services requires a high priority. Without it, it will not be possible to help those in need. Working in extremely difficult conditions, in a sense of high risk, additionally reinforced by many stress factors, indicates an urgent need to provide mental support, which should be implemented both by the management of the entities and the external environment. An important element of it is social support, which is a significant work resource [15] and has a positive impact on mental health. Support from friends and co-workers can reduce burnout by mitigating loneliness [16]. Support should be organized in such a way that employees try to constructively discuss problems, avoiding negative forms of communication. Psychiatrists themselves report that factors of the work environment, more than personal factors, play a greater role in the development of burnout [17]. This clearly shows that employees expect effective labor resources. Loneliness is related to seniority. Doctors with more experience were less likely to see themselves as lonely [11]. The aim of our study was to look at the phenomenon of occupational burnout in connection with the loneliness and complaining.

We stated a hypothesis that loneliness and complaining contributed to burnout. We assumed that some of the staff experience the problem of burnout. In our study, we hypothesized that there was no association between burnout and sociodemographic variables.

Loneliness and complaints of medical personnel in psychiatric practice are not well documented in the literature. There is no evidence of whether, and if so how, they are related to burnout. This problem appears especially when hospitals are overloaded with COVID-19. Our study is an important attempt to establish the correlation between loneliness experienced by psychiatric employees and individual tendency to complain and professional burnout. Results of our research can provide a valuable guidance for management improving the work in case of the environment of psychiatric care professionals.

## 2. Materials and Methods

### 2.1. Study Participants

A total of 265 medical employees from two psychiatric hospitals in the Pomeranian Voivodeship participated in the study. These were: the Provincial Psychiatric Hospital in Gdansk and the Hospital for the Nervous and Mentally Ill in Starogard Gdanski. The management of the institutions obtained consent to conduct the study. The total number of health professionals working in the two hospitals included in the study was 860. In the first hospital, out of 265 medical workers, 93 people applied for the study, of which 11 questionnaires were rejected due to incomplete answers. In the second hospital, out of 595 medical employees, 207 employees expressed their willingness to participate in the study, 183 of whom were finally qualified. All employees working in the hospital wards took part in the study. A written invitation was forwarded to the ward managers and then given to the employees. Afterwards, we contacted the employees who expressed their willingness to participate in the survey. The questionnaires were left in each ward. Only those willing took part in the study. An invitation to participate in the study was enclosed with the questionnaire. The subject of the study and information on the anonymity of the participants were given. All the participants gave their informed consent to participate in the study.

### 2.2. Study Questionnaire

The study was designed as a questionnaire survey and consisted of 4 sections with a total of 66 questions. The following is a detailed description of part of the questionnaire:

(1) Self-designed questionnaire—concerning socio-demographic data (gender, profession, work experience, education), and 6 questions concerning issues related to the organization of work during the pandemic of COVID-19 (assessment of the degree of organization of work in a team, assessment of team relations, the amount of support received from direct management, the amount of support received from the management of the facility and the degree to which teammates comply with the procedures and guidelines related to protecting health against COVID-19). The questionnaire consisted of 10 questions.

(2) The Oldenburg Burnout Questionnaire—OLBI is a tool that allows you to measure occupational burnout [7]. The questionnaire uses the two-factor concept of occupational burnout. The first factor is broadly defined exhaustion (taking into account its emotional, cognitive and physical components). Example of questions in this section are: “There are days when I feel tired before I arrive at work” or “During my work, I often feel emotionally drained”. The second component is distance from work, called disengagement, for example, in a form of a statement such as: “I feel more and more engaged in my work” or “Lately, I tend to think less at work and do my job almost mechanically”. The questionnaire consists of 16 questions. The questions were answered on a 4-point scale (1—I strongly agree, 2—I agree, 3—I disagree, 4—I strongly disagree) [18]. The questionnaire, both in its original and Polish version, is characterized by good psychometric properties. Cronbach’s alpha coefficient was calculated to assess internal consistency of the questionnaire in our study. Cronbach’s alpha was also good (α = 0.81).

(3) The De Jong Gierveld scale for measuring the sense of loneliness [19] and its Polish adaptation [20] allows to measure the perceived loneliness both in individual diagnosis and in scientific research. The scale contains a total of 11 items, most of which are formulated negatively, i.e., it allows to measure the dissatisfaction with social contacts (e.g., “I lack other people’s company”), and some positively, i.e., it measures satisfaction with social contacts (e.g., “on friends whenever I need it ”). The responses were rated on a 5-point scale (1—definitely yes, 2—yes, 3—neither yes nor no, 4—no, 5—definitely no). This questionnaire is also characterized by appropriate psychometric properties in both versions (original and adapted to Polish conditions). In this study, Cronbach’s alpha for this scale could be described as satisfactory (α = 0.72).

(4) The questionnaire of the content of daily conversations (Complaint Questionnaire) was designed by Bogdan Wojciszke and Wiesław Baryła [13]. We received it from the authors with permission to use it and their description of satisfactory psychometric characteristics. The Cronbach’s alpha for this questionnaire in this study was also good, α = 0.89. The questionnaire consists of 29 items and allows to measure the tendency to complain by answering the questions about how often the respondents talk about the mentioned topics. Some of the topics presented are positive, e.g., in the question about the frequency of talking about human kindness. Most of them deal with topics that are commonly associated with complaining: increasing crime, the heartlessness of officials and human indifference. Responses were rated on a 5-point scale (1—never, 2—rarely, 3—sometimes, 4—often, 5—very often). The questionnaire produces one overall score that is an indicator of a person’s propensity to generally complain.

### 2.3. Statistical Methods

All statistical analyses were performed with the Statistica 12.0 (StatSoft, Kraków, Poland). The study used parametric methods due to appropriate size of the study group, normal distribution of values and the continuous character of the variables. The Pearson correlation coefficient was used to measure the interrelationships between the variables. Differences between groups were tested using the Student’s *t*-test or one-way analysis of variance. Regression analysis (multiple regression type) was also performed.

## 3. Results

The completed questionnaires were returned by 265 (88%) respondents. Our study included medical personnel who have direct and closest contact with the patient on a daily basis. The detailed division of respondents by occupation is as follows: doctors (19.2%), nurses (69.8%), paramedics (4.9%), medical caregivers (5.7%). Most of the participants were female (82%) with long professional experience. Almost 60% of the studied group had higher education. The socio-demographic characteristics of the group are presented in Table 1.

There were no statistically significant connections between the group of socio-demographic variables and occupational burnout. There were no significant statistical differences between men and women in terms of exhaustion (t = 1.93, *p* = 0.06, df = 262), also in terms of disengagement (t = −0.42, *p* = 0.67, df = 262). Additionally, in the area of education, no statistically significant differences were observed between the two main groups in our study, one with higher and one with secondary education in terms of exhaustion (t = −0.84, *p* = 0.4, df = 262), or disengagement (t = −1.67, *p* = 0.09, df = 262). There were also no statistically significant differences in the context of exhaustion (F (5, 259) = 1.79, *p* = 0.11) and disengagement (F (5, 259) = 1.18, *p* = 0.32) between people working in different professions (doctor, nurse, paramedic, caregiver) within the unit of a psychiatric hospital.

The performed regression analysis (multiple regression type, all predictors entered simultaneously) allowed to obtain two statistically significant models, thanks to which it is possible to identify variables that are predictors of fatigue and disengagement. The regression model for exhaustion is presented in Table 2.

Loneliness (β = 0.20, *p* < 0.001) and complaining (β = 0.35, *p* < 0.001) proved to be statistically significant predictors of exhaustion. Work experience was the only non-significant predictor of fatigue. The whole model is statistically significant (F (3.261) = 20.12, *p* < 0.00001) and allows explanation of about 18% of the exhaustion variance (adjusted R^2^ = 0.178). The presence of a feeling of loneliness and complaining about many different topics allows us to predict severe exhaustion in psychiatric wards.

Another model containing predictors of disengagement is presented in Table 3.

The model is also statistically significant (F (3.261) = 10.37, *p* < 0.001) and allows to predict approximately 10% of the variance of disengagement (adjusted R^2^ = 0.96). In this case, significant predictors of disengagement turned out to be work experience (β = −0.13, *p* < 0.001), also loneliness (β = 0.13, *p* = 0.01) and complaints (β = 0.25, *p* < 0.001). In this case, the feeling of loneliness and intense complaining also allow to predict the lack of commitment to work. In the case of this model, it is possible to predict the lack of commitment based on the length of service—the longer the employee’s work experience, the smaller the chance of disengagement (employees with shorter work experience are less involved).

Further analysis involved establishing associations between loneliness and complaining and other variables. Significant statistical differences were revealed between women and men (t = 1.98, *p* = 0.04). Women (M = 2.86, SD = 0.57) are more likely to complain than men (M = 2.67, SD = 0.57). In case of this difference, we achieved Cohen’s *d* = 0.33, which means that the effect size is small. Additionally, complaining correlates in a statistically significant way with direct support from management (the less support, the greater the complaint).

Loneliness turned out to be related to organizational and team variables. High loneliness correlates in a statistically significant way with worse organization of work, also less support from administration management, management, worse working atmosphere (team relations) and surprisingly with more irresponsible attitudes of colleagues in relation to compliance with procedures and guidelines related to health protection against COVID-19. All results are presented in Table 4.

## 4. Discussion

Our research aims to examine the relationship between loneliness, complaining and lack of engagement, and to show their impact on burnout. We examined the relationships between socio-demographic variables (gender, occupation, seniority, education) and the phenomenon of occupational burnout. The analysis showed that these variables did not affect occupational burnout in the respondents. Gender was not significantly related to burnout. Confirmation of the lack of relationship between gender and the studied aspects can be found, among others, in the Bijari [21] and Dinibutun studies [22], which confirm the comparable level of burnout in both men and women. On the other hand, different results confirming the relationship between gender and exhaustion are given by Adam, who stated that women experienced higher emotional exhaustion [23], and Schadenhofer, who showed that gender had a different effect on the frequency of exhaustion [24]. Our study indicates that education did not affect exhaustion and lack of engagement, which is consistent with Oyefeso’s research [25]. Tan presents a different position on the subject, explaining the result with probably higher responsibility at work [26].

This study found no correlation between occupation and exhaustion and lack of engagement. Different positions can be found in the literature. A study of 200 employees showed that burnout was worse in nurses than in physicians, with both nurses and physicians having a higher level of burnout compared to other employees [27]. Mental health professionals may be more at risk of burnout than colleagues in other fields [28]. A study of psychiatrists in Milan confirm this assumption. A total of 49% of respondents [29] obtained a high level of emotional exhaustion.

The results of the current study showed that loneliness and complaining were important predictors of exhaustion. An employee who experiences exhaustion begins to feel more and more lonely. They begin to isolate themselves from others, their workshop slowly deteriorates and their professional activities become more and more difficult. When there is no support in the immediate surroundings, the feeling of loneliness grows, leading to even greater exhaustion over time. Experiencing more and more discomfort, they begin to critically evaluate situations that have so far been positive or indifferent. Criticism increases. They begin to complain. The more tired they are, the more they complain. Loneliness in the workplace is noticed by colleagues and significantly affects the efficiency of work and relationships in the team by weakening social relationships in the group. Thus, loneliness leads to withdrawal and reduction in job productivity [30]. In process of time, a person experiencing loneliness begins to feel worse, which is a negative consequence of loneliness [31]. In their research, Killgore and colleagues show that loneliness is getting worse during the current pandemic [32].

The results of the study are confirmed in the Ofei-Dodoo research, which indicates the correlation of a high level of exhaustion with a sense of loneliness [10]. In another study, Rogers confirms the relationship between loneliness and burnout, indicating that higher levels of loneliness were significantly associated with higher burnout [16]. It should also be remembered that the emotional exhaustion experienced by the employee will have an influence on their private life, over time impeding the fulfillment of Zhang’s family tasks and roles [33].

Dinibutun, in his work, states that there is a significant difference in exhaustion between nurses and doctors [22]. It may be because of the nature of the job. It is the nurse who is closest to the patient, spending a lot of time with them in direct contact. When the patient is agitated, the nurse must act quickly, trying to ensure the safety of both herself and the patient. It is very difficult, it causes a lot of emotional tension and ultimately leads to exhaustion.

Complaining means focusing on the negative aspects of the surrounding reality; more importantly, communicating using such a pattern. In a long-term complaining process, it may happen that the subject of the complaint is anything for the sake of the idea of complaining.

An extremely important issue is the impact of the behavior of people experiencing burnout on the relationships in the team. This problem is highlighted in Maslach’s research, emphasizing that employee burnout may have a negative impact on other team members; consequently, leading to their distance from work and worsening social relations in the team [5]. Therefore, both difficult emotions and a lack of commitment to work are spilled to some extent. It does not take a long-time perspective for the quality of care to deteriorate.

Loneliness is naturally associated with a tendency to withdraw. The employee starts to move away from others, communication and performance of tasks may deteriorate. The fact that we work as a team in hospitals may turn out to be beneficial in this case. You can quickly notice a person who starts to move away from the group. The first source of support for an employee may be a group of colleagues at work. They are important in providing support. The research of many authors indicates the important role of social support as an element preventing burnout. The support offered by both colleagues and friends is of great importance, as confirmed by their research by Aronsson [34] and Mohindra [2].

Patients with mental health problems may not be able to comply with a sanitary regime. Poor self-care capacity and insufficient insight are factors that significantly hinder cooperation in this regard [35]. Many of the existing rules must be changed. The long-term promotion of group activities of patients should be currently slowed down by proposing to keep social distance. The problem, however, is that this is the opposite course of action. According to Arango, we have never encouraged patients to distance themselves [35]. Studies of patients experiencing psychosis show that they badly need emotional support from their loved ones. This need is confirmed by 91% of respondents in the Lahera study [36]. Due to the pandemic, large visiting restrictions are, as it were, going against. Additionally, although patients, understanding the necessity to introduce restrictions, try to comply with them, it is very difficult for them because of the separation from their loved ones.

The presence of all these difficulties and their mutual penetration, strengthened in the pandemic era, with care for their own health and safety, causes employees to gradually show an increasing lack of commitment at work. The superior goal of helping the patient may turn out to be disastrous for the helper. Significant predictors of disengagement are seniority, loneliness and complaining. The length of the work experience allows to forecast the level of disengagement. Employees with longer experience show less disengagement. We can find confirmation of seniority in the literature, where younger people are more likely to be exhausted [25]. In other studies, the author shows that greater age and experience were associated with less burnout [37]. More experience goes hand in hand with more practice and some kind of distance, which can be important when dealing with minor problems and failures at work. Another analysis was to establish the links between loneliness and complaining and other variables. According to the results, women are more prone to complaining. This may be related to greater sensibility to stress [38]. Women are more sensitive. As mothers, they strive for the proper development of their children and the safety of the whole family. Bearing in mind that human resources are the most valuable asset of an organization [39], management should make every effort to provide a supportive work environment. The key importance of support from the employer is mentioned by many authors in their research. Belfroid et al. emphasize the importance of the quick availability of a superior in a difficult situation [40]. In turn, Kang et al. emphasize the importance of clear communication in the process of supporting employees [41].

A clear division of duties, application of procedures and compliance with the rules contribute to shaping a proper and safe atmosphere in the team. The support needs of employees are individual and depend on many factors. Nevertheless, employer support seems to be a key element in the fight against burnout.

Caring for a safe work environment includes various aspects, both physical, in the form of providing personal protective equipment, and emotional, in the form of supporting employees and paying attention to their needs [33]. The control also contributes to reducing tension, and thus burnout [42], as it allows obtaining substantive information on the tasks performed. Our analysis is confirmed by studies by other authors showing that a high level of support protects against exhaustion [34]. In times of a pandemic, concern for the psychological safety of employees becomes of particular importance. Wu, in his research, shows that if workers feel that they will be supported in case of a disaster, they will be more resilient [43]. Looking at this issue from the other side, it should be stated that if we do not provide support to employees, the degree of complaining and the feeling of loneliness will increase. Additionally, this can lead to leaving the profession. Interesting results on this topic were presented by Shah, who showed that among nurses who reported resignation from the profession due to burnout, a high percentage indicated a stressful work environment as the reason [44]. Our study has some limitations. One of the limitations of our study is that it was conducted on a small group of regional employees. The study covered the staff of psychiatric hospitals in the Pomeranian Voivodeship. In the future, it is worth extending the survey to at least the entire country to make comparisons with surveys carried out in other countries. Another limitation of the project is the fact that the study was carried out during a pandemic, i.e., a time when health care staff was particularly burdened. However, it seems to us that psychiatric staff will long struggle with overload, also in a post-pandemic society.

## 5. Conclusions

Our study shows that loneliness, complaining and disengagement are significant predictors of psychiatric staff exhaustion. It is very important to create a friendly work environment. Support should take into account both an individual and a team approach. Loneliness, complaining and disengagement may be changed by organizing team meetings and staff training sessions to create a community. Such meetings could be enriched with psychoeducational elements indicating the psychological consequences of complaining and various affirmation techniques. It is important that it is systematic and allows flexible adaptation to the employee. The management of the institutions should take into account the prevention of infodemia, taking care of the substantive and safe transmission of information. Strong leadership and a clear management structure are a protective factor in this case. Moreover, it is essential to systematically monitor the phenomenon of burnout in individual professional groups.

Our study shows that loneliness and complaining are important concepts that predict significant outcomes in the area of occupational burnout in mental health care employees. It is worth undertaking further research on this issue in order to better understand the various interactions between loneliness, complaining and burnout. The obtained results will allow for taking effective measures in the field of burnout prevention. Problems with recruiting and maintaining medical staff, present in most facilities for many years, require management to pay attention to the employee’s satisfaction, health and job satisfaction. Professional activity means that we spend a significant part of our lives at work. Therefore, it is necessary to ensure that the time spent there is fully satisfactory for the employees, which will translate into the satisfaction of our customers.

## Figures and Tables

**Table 1:** Socio-demographic characteristics of the study group.

Socio-Demografic Variables	Employees (*N* = 265)
Gender (*N*; %)	
Women	217 (82%)
Men	47 (18%)
Profession	
Doctor	51 (19.2%)
Nurse	185 (69.8%)
Paramedic	13 (4.9%)
Caregiver	15 (5.7%)
Other	1 (0.4%)
Job seniority	
Up to 2 years	14 (5%)
2–10 years	51 (19%)
10–20 years	53 (20%)
Over 20 years	147 (55%)
Education	
Secondary vocational education	106 (40%)
Higher	158 (59.6%)
Other	1 (0.4%)

**Table 2:** Regression model for the exhaustion variable with significant predictors in the group of employees of psychiatric wards.

Coefficient	Estimate β	t	*p*
Job seniority	−0.09	−1.69	
Loneliness	0.20	3.63	*
Complaining	0.35	6.32	*

* *p* ≤ 0.001.

**Table 3:** Regression model for the variable of disengagement with significant predictors in the group of employees of psychiatric wards.

Coefficient	Estimate β	t	*p*
Job seniority	−0.13	−1.69	**
Loneliness	0.13	3.63	*
Complaining	0.25	6.32	**

* *p* ≤ 0.01, ** *p* ≤ 0.001.

**Table 4:** Correlations between loneliness and complaining and organizational and interpersonal variables related to the syndrome (Pearson’s correlation coefficient).

Variables	Loneliness	Complaining
Organization of team work	0.14 *	0.06
Relationships in the team	0.24 *	0.06
Administration support	0.19 *	0.18 *
Management support	0.23 *	0.22 *
Staff attitudes towards COVID-19	0.20 *	0.16

* *p* < 0.05.

## Data Availability

The datasets used and/or analyzed during the current study are available from the corresponding author on reasonable request.
